# Precipitating Factors, Complications, and Outcomes of Diabetic Ketoacidosis (DKA) in Adults and Pediatrics: A Descriptive Study from Two Tertiary Centers in Riyadh, Saudi Arabia

**DOI:** 10.3390/jcm14238505

**Published:** 2025-11-30

**Authors:** Osamah M. Alfayez, Ghazwaa G. Almutairi, Shahad B. Alqudhibi, Mayyadah A. Alnefaie, Sadeem D. Alshehri, Ruba K. Alzaidi, Dona M. Alassiri, Lama R. Alkhathran, Dalal A. Alabdulkarim, Majed S. Al Yami, Sultan M. Alghadeer, Omar A. Almohammed

**Affiliations:** 1 Department of Pharmacy Practice, College of Pharmacy, Qassim University, Buraydah 52571, Saudi Arabia; 2 Department of Clinical Pharmacy, College of Pharmacy, King Saud University, Riyadh 11451, Saudi Arabia; 446200064@student.ksu.edu.sa (G.G.A.); shah-od1@hotmail.com (S.B.A.); salghadeer@ksu.edu.sa (S.M.A.); 3 Department of Pharmacy Practice, College of Pharmacy, King Saud bin Abdulaziz University for Health Sciences, Riyadh 11481, Saudi Arabiayamim@ksau-hs.edu.sa (M.S.A.Y.); 4 Pharmaceutical Care Service, Ministry of the National Guard-Health Affairs, Riyadh 11481, Saudi Arabia; 5 King Abdullah International Medical Research Center, Riyadh 11481, Saudi Arabia; 6 Pharmacoeconomics Research Unit, College of Pharmacy, King Saud University, Riyadh 11451, Saudi Arabia

**Keywords:** diabetic ketoacidosis, Saudi Arabia, precipitating factors, non-compliance, electrolyte disturbances, hyperkalemia, hypokalemia, acute kidney injury

## Abstract

**Background:** Diabetic ketoacidosis (DKA) is a serious acute complication of diabetes mellitus (DM) associated with significant morbidity, mortality, and healthcare burden worldwide. This study aimed to investigate population descriptors and clinical outcomes among adult and pediatric patients admitted with DKA at two tertiary medical centers in Riyadh, Saudi Arabia. **Methods:** We conducted a retrospective observational study that included adult and pediatric (≤15 years) patients admitted to emergency departments (EDs) and received care for DKA between 2018 and 2021. DKA severity was defined according to the American Diabetes Association (ADA) criteria, which rely on arterial/venous pH and serum bicarbonate (with anion gap supportive), as follows: mild (pH 7.25–7.30; HCO_3_^−^ 15–18 mmol/L), moderate (pH 7.00–7.24; HCO_3_^−^ 10–15 mmol/L), and severe (pH < 7.00; HCO_3_^−^ < 10 mmol/L). Data were extracted from electronic medical records and analyzed descriptively. **Results:** A total of 373 patients were admitted to the EDs and received treatment for DKA throughout the study period. Adults constituted 71.6% (267/373), while children represented 28.4% (106/373) of the patients; the majority of adults (74.2%) had Type 1 DM (T1DM), while all pediatric patients had T1DM. More than half of the adult presentations met the criteria for severe DKA (55.8%; 149/267), whereas pediatric cases were most commonly moderate in severity (41.5%; 44/106). The most common precipitating factors across both age groups of patients with diabetes before the index DKA event were non-compliance with therapy and infection. Both groups demonstrated typical biochemical features of DKA, although pediatric patients presented with slightly lower bicarbonate and higher anion gaps (slightly greater metabolic acidosis) but with similar hydration status. Regarding patients’ outcomes, hyperkalemia was identified in 23.6% of adults and 24.5% of pediatric patients, while hypokalemia was documented in 20.2% of adults and 24.5% of pediatric patients, and adult patients experienced more acute kidney injuries than the other cohort (5.2% vs. 1.9%). In-hospital mortality was 0.8% (3/373) among all adults. Although pediatric patients experienced faster DKA resolution (median = 16.5 h; IQR, 11.7–25.8) compared to adult patients (23.7 h; 16.2–36.9), they had a longer hospital stay compared to adult patients, and a significant majority required ICU care (50.9%) at some point during their care. **Conclusions:** The increasing prevalence of DM in Saudi Arabia, especially among the youth, would lead to an increase in DKA burden unless effective preventive measures are taken. This study demonstrated that preventable causes, such as non-compliance with therapy and infection, were responsible for the high admission rates. Thus, comprehensive outpatient care can help strengthen care continuity and help decrease the burden on emergency and inpatient services.

## 1. Introduction

Diabetic ketoacidosis (DKA) remains one of the most serious acute complications of diabetes mellitus (DM). It is associated with morbidity, mortality, and healthcare burden worldwide. DKA continues to occur across all age groups and often results in hospitalizations [1,2,3,4]. In Saudi Arabia, diabetes prevalence has risen steadily over the past two decades, including type 1 diabetes mellitus (T1DM) [5,6,7,8]. This increase, particularly among younger populations, has translated into a growing incidence of DKA presentations.

DKA is an acute metabolic emergency characterized by a blood glucose level > 11 mmol/L, a serum bicarbonate level < 15 mmol/L, and a venous pH < 7.3, in addition to the presence of symptoms such as glucosuria, ketonemia, and ketonuria [1,2]. If it is not timely recognized and treated, DKA can lead to serious complications such as metabolic imbalance, hyperkalemia, and life-threatening complications like cerebral edema and acute kidney injury (AKI) [9].

Previous studies from various regions of the Kingdom have shown that a significant proportion of DKA cases are precipitated by preventable factors. These factors often include missed insulin doses, non-compliance with therapy, and infections [10,11,12,13,14]. However, most existing reports have examined either pediatric or adult populations separately, with limited comparative data exploring differences in severity, biochemical profiles, and clinical outcomes between the two groups.

Understanding these variations is critical for tailoring preventive strategies. It will also aid in optimizing inpatient management and improving post-discharge education. Therefore, this study aimed to investigate population descriptors and clinical outcomes among adult and pediatric patients admitted with DKA at two tertiary medical centers in Riyadh, Saudi Arabia.

## 2. Methods

### 2.1. Study Design, Setting, and Population

We conducted a retrospective observational study to explore population descriptors and clinical outcomes among adult and pediatric patients admitted with DKA. This study was conducted at King Saud University Medical City (KSUMC) and King Abdulaziz Medical City (KAMC) in Riyadh, Saudi Arabia. All patients (pediatric and adult) admitted to emergency departments (ED) and received care for DKA between 2018 and 2021 were included in the study. These patients also had a previously confirmed diagnosis of T1DM or Type 2 DM (T2DM) or were newly diagnosed with diabetes at the time of admission for the index DKA event. Patients were categorized according to their age at the time of presentation to the emergency department: those aged 15 years or younger were classified as pediatric patients, while those older than 15 years were classified as adults; note that this age classification was independent of the patient’s age at diabetes onset.

DKA was defined according to the American Diabetes Association (ADA) guidelines and the Joint British Diabetes Society (JBDS) criteria as follows: the presence of (1) hyperglycemia, typically plasma glucose > 11.1 mmol/L (> 200 mg/dL); (2) metabolic acidosis with venous or arterial pH < 7.30 and/or serum bicarbonate < 15 mmol/L; and (3) evidence of ketonemia or ketonuria [1,2]. The DKA severity for adults was classified based on the ADA guidelines as follows: mild (pH 7.25–7.30; HCO_3_^−^ 15–18 mmol/L), moderate (pH 7.00–7.24; HCO_3_^−^ 10–15 mmol/L), or severe (pH < 7.00; HCO_3_^−^ < 10 mmol/L) [1]. Pediatric patients were classified based on the International Society for Pediatric and Adolescent Diabetes and American Academy of Pediatrics (ISPAD/AAP) guidelines as follows: mild (pH 7.20–7.30; HCO_3_^−^ 10–15 mmol/L), moderate (pH 7.10–7.19; HCO_3_^−^ 5–9 mmol/L), or severe (pH < 7.10; HCO_3_^−^ < 5 mmol/L) [3]. All patients met the ADA criteria for DKA with metabolic acidosis and hyperglycemia, while ketonemia/ketonuria was documented in nearly all cases. However, when ketonemia/ketonuria was not available or not documented, we relied on physician notes to diagnose DKA. Cases with isolated lactic acidosis (elevated lactate with acidosis but no ketonemia/ketonuria) were excluded. Patients who did not meet the pre-specified criteria for the DKA diagnosis, did not have or were not diagnosed with DM, or had non-diabetic ketoacidosis were excluded.

### 2.2. Data Collection and Variables

All patients’ information was collected from the electronic medical records at the two centers and analyzed. The data from this study was extracted independently using the Research Electronic Data Capture (REDCap) data extraction sheet. The data obtained included demographic and physiological variables such as age, weight, height, and body mass index (BMI). Each patient’s medical history was reviewed, including family history of diabetes, diabetes type, diabetes onset, diabetes-related complications, and the presence of comorbidities before admission for the DKA event. The onset of diabetes in this study was determined by reviewing patients’ medical records. New-onset diabetes was defined as a first diagnosis of diabetes during the index DKA admission, while pre-existing diabetes referred to patients with a prior documented diagnosis of diabetes before the index DKA presentation.

The patients’ mental states and vital signs, as well as laboratory findings such as blood glucose from a fingerstick and lab (mmol/L), arterial blood gas parameters including pH and bicarbonate (mmol/L), and urine ketones, were evaluated upon admission. Also, electrolytes, such as potassium (mmol/L), sodium (mmol/L), corrected sodium (mmol/L), and chloride (mmol/L), and renal function tests, including serum creatinine (umol/L), creatinine clearance (ml/min), and blood urea nitrogen (mmol/L), were analyzed. In addition, the anion gap and serum osmolarity were calculated based on these laboratory values.

Among patients previously diagnosed with diabetes, the reasons for DKA admission were identified based on the treating physician’s documentation in the medical record, which included non-compliance, inadequate or insufficient insulin administration, insulin pump malfunction, or insulin shortage. Likewise, predisposing factors such as infections, acute pancreatitis, trauma, myocardial infarctions, cerebrovascular accidents, and alcohol or drug abuse were also recorded, as documented in the medical records by the treating physician. Also, their medication history for diabetes management before hospitalization was obtained, including whether patients were on insulin therapy, hypoglycemic agents, or both.

Clinical outcomes were obtained, such as time of DKA resolution, complications during hospitalizations (including hyperkalemia (K > 5.2 mmol/L), hypokalemia (K < 3.3 mmol/L), hypoglycemia (glucose < 4.0 mmol/L), AKI, pulmonary edema, and cerebral edema), in-hospital mortality, and length of stay (LoS) in hospital. Time to DKA resolution was defined as a reduction in serum glucose to less than 200 mg/dl (<11 mmol/L) with improvement in at least two of the following parameters: serum bicarbonate ≥ 15 mmol/L, anion gap ≤ 15 mmol/L, and venous pH more than 7.3.

### 2.3. Statistical Analysis

Patients’ characteristics were summarized using descriptive statistics, including the mean and standard deviation (SD) for normally distributed continuous data or the median and interquartile range (IQR) for non-normally distributed data. While categorical data were summarized using frequencies with percentages. Additionally, a subgroup analysis was performed on all patients, including patients with documented diagnoses of T1DM or T2DM, to present the study findings according to diabetes type. Data were gathered using the Research Electronic Data Capture (REDCap^®^) software (Vanderbilt University, Nashville, TN, USA). The data collected was cleaned and managed using Microsoft Excel version 2019 (Microsoft Corp., Redmond, WA, USA), while the SAS software, version 9.4 (SAS Institute Inc., Cary, NC, USA) was used for all statistical analyses.

### 2.4. Ethical Consideration

Institutional review board approvals were obtained from the participating medical centers: KAMC/King Abdullah International Medical Research Center (KAIMRC) and KSUMC.

## 3. Results

### 3.1. Study Population and Baseline Characteristics

A total of 373 patients were admitted to the ED and received treatment for DKA throughout the study period. The majority were adults (71.6%; 267/373), with a median age of 24.3 years (IQR, 17.5–39.2). The median age of pediatric patients was 12.9 years (11.3–13.9). Regarding a family history of diabetes, 5.6% of adults (15/267) had relatives with T1DM, while 9.7% (26/267) had relatives with T2DM. Among pediatrics, 20.8% (22/106) had relatives with T1DM, while 18.9% (20/106) had relatives with T2DM. Regarding the onset of diabetes, 88.8% of adult cases (237/267) had pre-existing diabetes, whereas 11.2% (30/267) had a new onset of diabetes. Similarly, among pediatric patients, 79.2% of cases (84/106) had pre-existing diabetes, while 20.8% (22/106) had a new onset of diabetes. T1DM was the most prevalent type among both adult and pediatric patients. These data are summarized in Table 1.

In adults, the most prevalent comorbidities were hypertension (18.0%; 48/267) and dyslipidemia (17.6%; 47/267), while the prevalence of comorbidities was generally low in pediatric patients; only 3.8% of pediatric patients (4/106) had asthma, while 7.5% of them (8/106) had different comorbidity types than the ones listed in Table 1. While all pediatric patients and most adults (82.5%; 220/267) had no documented diabetes-related complications, the most common complication in adults was diabetic retinopathy (5.6%; 15/267), followed by nephropathy (3.7%; 10/267) and diabetic foot injury or amputation (3%; 8/267).

### 3.2. Severity Classification of DKA Cases

Regarding the severity of DKA cases, more than half of the adult patients presented to the ED with severe DKA (55.8%; 149/267), while the remaining had mild (15.4%; 41/267) or moderate DKA (28.8%; 77/267). On the other hand, about one-third of the pediatric patients had severe DKA (34.9%; 37/106), while 23.6% of them presented with mild DKA, and 41.5% (44/106) had moderate DKA. The classification of patients based on the severity of their DKA, along with their values under each laboratory parameter, is summarized in Table 2.

### 3.3. Reasons for DKA Admission and Predisposing Factors (For Patients with Previously Diagnosed DM)

The most frequently documented reason for these ED admissions among adult patients was non-compliance with therapy, accounting for 61.6% (146/237) of cases, while among pediatric patients, this reason accounted for 48.8% (41/84). Subsequently, 5.1% (12/237) of adults and 8.3% (7/84) of pediatric patients had inadequate or insufficient insulin therapy. In addition, insulin pump malfunction and a lack of insulin therapy were other reasons for DKA, albeit with less prevalence, as illustrated in Table 3.

Additionally, several documented predisposing factors for DKA events were identified. Infection was the most common contributor, affecting 20.6% (66/321) of patients with documented DM before the index DKA event. Other factors were much less common and seen predominantly in adults, including acute pancreatitis, severe burn or trauma, alcohol abuse, cerebrovascular accidents, drug abuse, and myocardial infarctions. Because patients with a new onset of DM had no external precipitant, we presented precipitating factors and reasons for admission while excluding patients with a new onset of DM (Table 3).

### 3.4. Medication History and Insulin Regimens Before Admission

Insulin injections/pens were the most used therapy in both groups, with 77.9% (208/267) of adults and 75.5% (80/106) of pediatric patients receiving insulin injections/pens (Table 4). Insulin pump use was low in both adult and pediatric patients, at 4.5% (12/267) and 3.8% (4/106), respectively. Among the types of insulin used, rapid-acting insulin and long-acting insulin were the most used among the entire cohort. Regarding other non-insulin hypoglycemic agents, 8.6% of adults (23/267) were on biguanides, while other non-insulin therapies were less commonly used. Glucagon-like peptide-1 receptor agonists (GLP-1RAs) were used in both groups: 2.8% in pediatric patients (3/106) and 0.4% (1/267) in adults. A considerable proportion of patients had no documented medication history (adults: 13.1%, 35/267; and pediatrics: 20.8%, 22/106), and most were not previously diagnosed with DM.

### 3.5. Mental Status and Laboratory Values at Admission

About 85.4% (228/267) of adult patients and 84.0% (89/106) of pediatric patients had an alert mental status at the time of admission. Furthermore, 13.5% (36/267) of adults had an alert/drowsy mental status compared to 16.0% (17/106) of pediatric patients. Only three adult cases were unconscious and in coma at admission (1.1%; 3/267). Table 5 summarizes the baseline mental status and laboratory values of these patients upon admission.

The laboratory values at admission were consistent with the typical biochemical profile of DKA in adult and pediatric patients. The patients presented significant hyperglycemia, with a median glucose level from finger sticks of 23.1 mmol/L (16.7–29.5) in adults and 23.0 mmol/L (18.3–27.0) in pediatric patients. Glucose levels from the laboratory were similar but slightly higher in adult and pediatric patients, at 25.9 mmol/L (19.6–32.4) and 24.5 mmol/L (16.9–28.8), respectively. The electrolyte profiles showed some differences between the adult and pediatric cohorts. The median serum sodium level was 133.0 mmol/L (130.0–136.0) in adults and 134.0 mmol/L (131.0–137.0) in pediatric patients. Moreover, adult patients had a median potassium level of 5.0 mmol/L (4.4–5.4), compared to 4.9 mmol/L (4.4–5.4) in pediatric patients.

The admission data revealed a state of metabolic ketoacidosis and hyperosmolality in both patient groups. Both groups had a median pH of 7.2 (7.1–7.3), but the median anion gap in pediatric patients was wider at 26.0 (21.0–30.0) compared to 23.0 (19.0–28.0) in adult patients. Serum osmolality was 304.0 mOsm/kg H_2_O (293.0–316.0) in adults and 298.0 mOsm/kg H_2_O (291.0–307.0) in pediatric patients. Positive urine ketones were documented in all pediatric patients and 93.3% (249/267) of adult patients. Regarding the renal panel, adult patients showed a median serum creatinine level of 97.0 μmol/L (76.0–127.0), a creatinine clearance (CrCl) level of 70.0 mL/min (51.5–89.5), and a BUN value of 5.4 mmol/L (4.1–7.0). In pediatric patients, the median values for serum creatinine, CrCl, and BUN were 81.0 μmol/L (69.0–97.0), 62.3 mL/min (46.8–83.2), and 5.4 mmol/L (3.8–6.3), respectively (Table 5).

### 3.6. Patients’ Outcomes

#### 3.6.1. Hospital Admission Location and Length of Stay

For adults, most patients (69.3%; 185/267) were admitted to general wards only, and a smaller portion of adults required ICU care, with 7.9% (21/267) admitted to the ICU alone and 12.7% (34/267) admitted to both general wards and the ICU during their course of care for DKA management. For pediatric patients, 42.5% (45/106) were admitted to general wards only, while 11.3% (12/106) were admitted to the ICU only, and 39.6% (42/106) were admitted to both general wards and the ICU. While 10.1% (27/267) of adult patients were discharged directly from the ED, only 6.6% (7/106) of pediatric patients were discharged from the ED without the need for hospitalization. Patient admission status is presented in Table 6.

When examining the LoS, the median LoS in the ED for the adult patients was 8.3 h (4.9–16.4), compared to 3.8 h (2.6–6.9) for the pediatric patients. The overall median LoS in the hospital was 2.0 days (1.3–3.5) for adults, and their median LoS in the general ward and the ICU were 1.5 days (1.0–3.0) and 1.2 days (0.5–2.2), respectively. For pediatric patients, the overall median LoS in the hospital was 2.1 days (1.5–3.0), and their median LoS in the general ward and the ICU were 1.5 days (1.0–2.3) and 0.9 days (0.6–1.4), respectively (Table 6).

#### 3.6.2. DKA Resolution

DKA resolution was documented in 83.9% (224/267) of adults and 78.3% (83/106) of pediatric patients during their hospitalization. The median time to DKA resolution was shorter for the pediatric patients (16.5 h; 11.7–25.8) compared to adult patients (23.7 h; 16.2–36.9), as illustrated in Table 6.

#### 3.6.3. Treatment-Related Complications

Hyperkalemia (K > 5.2 mmol/L) was the most prevalent complication among these patients during DKA management; it occurred in 24.5% (26/106) of pediatric patients and 23.6% (63/267) of adult patients. Next was hypokalemia (K < 3.3 mmol/L), which occurred in 20.2% (54/267) of adults and 24.5% (26/106) of pediatric patients. Hypoglycemia (glucose < 4.0 mmol/L) was recorded in 9.7% (26/267) of adults and 5.7% (6/106) of pediatric patients. However, AKI (4.3%; 16/373), pulmonary edema (0.5%; 2/373), and cerebral edema (0.5%; 2/373) were relatively rare in both groups. Lastly, three adult patients (1.1%) died during their hospitalization after a long hospitalization period; this was mainly due to reasons other than DKA. Complications developed during hospitalizations are presented in Table 6.

### 3.7. Subgroup Analysis Based on Patients’ Documented Type of DM

A total of 368 patients with documented T1DM or T2DM were included in this subgroup analysis. The majority of the cohort had T1DM (82.6%; 304/368) and a median age of 16.7 years (IQR, 13.8–23), whereas patients with T2DM accounted for 17.4% (64/368) and had a median age of 55.2 years (IQR, 41.5–64.2). Patients with T2DM were more likely to have comorbid conditions, particularly hypertension and dyslipidemia. Regarding DKA severity among adults, the majority had severe DKA for both T1DM (59.1%; 117/198) and T2DM (46.8%; 30/64), whereas most pediatric patients, who all had T1DM, had moderate DKA (41.5%; 44/106).

For patients with documented T1DM and T2DM before the index DKA event, the most frequently documented reason for these ED admissions was non-compliance with therapy, accounting for 59.7% (157/263) and 51.8% (29/56) of patients, respectively. Moreover, several documented predisposing factors for DKA events were identified, with infections being the most common contributor for this DKA event in patients of both types, affecting 18.6% of patients with T1DM and 28.6% of patients with T2DM.

Regarding the medication history before admission, insulin injections/pens were the most used therapy in both groups (T1DM, 80.3% (244/304) and T2DM, 65.6% (42/64)). In contrast, 28.1% of patients with T2DM (18/64) were on biguanides, compared to 1.6% (5/304) of patients with T1DM, and other non-insulin therapies were less commonly used. At admission, laboratory values were consistent with the typical biochemical DKA profile for both types. The patients presented with substantial but comparable hyperglycemia, with a median glucose level (finger stick) of 23.0 mmol/L (17.3–28.4). Patients with T2DM had a higher median serum creatinine level compared to patients with T1DM (120.5 umol/L; IQR, 92.5–153.5 vs. 88.5 umol/L; IQR, 73.0–112.0).

Among patients with T1DM, 57.9% (176/304) were admitted to general wards only, while 31.3% (95/304) needed ICU care during their hospitalization, and the majority of patients with T2DM were admitted to general wards only (78.1%; 50/64). When examining the LoS, the median LoS in the ED for patients with T1DM was 5.9 h (3.7–14.3), compared to 9.9 h (6.6–15.7) for patients with T2DM. The overall median LoS in the hospital for patients with T1DM was 1.8 days (1.2–3.0), compared to 2.9 days (1.8–5.0) for those with T2DM. The median time to DKA resolution was comparable between the two subgroups, but it was shorter for patients with T1DM (21.4 h; IQR, 14.3–33.7) compared to those with T2DM (24.0 h; IQR, 16.0–38.0). Hyperkalemia was the most prevalent therapy complication among patients with T1DM during their DKA management; it accounted for 25.7% (78/304) of patients with T1DM and 15.6% (10/64) of patients with T2DM. Next was hypokalemia (K < 3.3 mmol/L), which occurred in 21.4% (65/304) of patients with T1DM and 21.9% (14/64) of patients with T2DM. The results discussed here are summarized in Tables S1–S6 of the Supplementary Materials.

## 4. Discussion

The aim of this study was to investigate population descriptors and clinical outcomes among adult and pediatric patients admitted with DKA. Among the cohort, new-onset T1DM was observed among the pediatric patients. The most common precipitating factors in patients with documented diabetes before the index DKA event across both age groups were non-compliance with therapy and infections. Adults constituted most of the cohort (71.6%), and more than half of the adult presentations met the criteria for severe DKA, whereas pediatric cases were most commonly moderate in severity. At presentation, both groups demonstrated typical biochemical features of DKA. Pediatric patients showed slightly lower bicarbonate levels and higher anion gap values, indicating somewhat greater metabolic acidosis but similar hydration status. Regarding patients’ outcomes, hypokalemia and hyperkalemia were frequently encountered during hospitalization in both age groups, and adult patients experienced more AKI. Pediatric patients experienced faster DKA resolution; however, they had a longer total hospital LoS, and a significant majority of pediatric patients required ICU care (50.9%) at some point during their stay.

The study cohort has some important similarities and differences compared to other studies conducted in Saudi Arabia. In our study, the median age of pediatric patients at presentation was 12.9 years, which is within the early adolescent period. This is likely attributed to our inclusion criteria, which included patients up to 15 years old in this age category. The mean age in other studies ranged from 6.9 years to 8.9 years [12,13,14,15]. On the other hand, the median age for the adult cohort is 24.3 years, which is on the younger side compared to other studies [10,11].

In our study, we have reported lower DKA at T1DM onset (20.8%) among pediatric patients compared to most studies conducted in Saudi Arabia (25.6 to 29.8%) [12,13,14,15]. However, this is in line with population-based studies conducted worldwide (19% to 40%) [16,17]. The median laboratory values at admission for pediatric patients showed moderate-to-severe DKA, which is consistent with other studies [12,15]. For instance, a study conducted in Jeddah, Saudi Arabia, by Alsolaimani et al. found that most pediatric patients presented with moderate (39.5%) or severe (29.3%) DKA [15]. Also, Babiker et al. reported that most of their pediatric patients had moderate DKA (67.1%) [12]. On the other hand, the adult cohort of our study presented with moderate DKA, which is consistent with the data from other centers, such as that reported by AlWahbi et al. and Alotaibi et al. [10,11].

In our study, non-compliance with therapy and infections were the most common precipitating factors for DKA in both pediatric and adult patients with previously documented DM before the index DKA event. This finding is in alignment with both the global scale and the context of Saudi Arabia. Two Saudi-based studies by Alotaibi et al. and AlWahbi et al. have reported both non-compliance and infections as the most common precipitating factors [10,11]. Similarly, a study from Qatar by Ata et al. found that non-compliance (28.4%) and infections (26.1%) were the top two documented triggers for DKA in adult patients [18]. Moreover, a Jordanian study has found that, in patients with recurrent DKA, 41% of cases were due to missed insulin doses, while 38% were due to acute illness (infection) [19]. Additionally, newly diagnosed patients with diabetes who present with DKA at onset represent a distinct clinical subgroup. Unlike DKA episodes in individuals with established diabetes, these cases typically occur without external precipitants such as infections or treatment non-compliance.

Almost all included patients fulfilled the ADA diagnostic criteria for DKA, including hyperglycemia, metabolic acidosis, and ketonemia or ketonuria. Mild hyperlactatemia can occur in DKA, but true metformin-associated lactic acidosis (MALA), characterized by isolated lactic acidosis without ketosis, was not identified. Although 23 adult patients were using metformin, all patients exhibited biochemical profiles consistent with DKA rather than MALA. Note that lactate data were incomplete; therefore, the ability to assess mixed acid–base disorders was limited in our case.

An important observation from our study was that pediatric patients showed a shorter median time to DKA resolution (16.5 h) compared with adults (23.7 h), as well as a slightly longer total hospital length of stay (2.1 vs. 2.0 days). The difference in resolution time may reflect variations in clinical characteristics or management practices between the two groups. The longer hospitalization among pediatric patients likely relates to the higher rate of ICU admission (50.9% vs. 20.6% in adults), suggesting that although biochemical correction appeared faster in children, their overall care required more intensive observation and supportive management. The observed differences in DKA resolution time and hospital stay may also be influenced by multiple factors, including patient heterogeneity, the clinical context, or random fluctuations. Therefore, these results should be interpreted cautiously as descriptive observations rather than definitive or causal group differences, given that no inferential statistical analyses were performed in this exploratory study.

It is important to note that, although both hospitals follow comparable DKA management protocols that were mainly based on the ADA, JBDS, and ISPAD/AAP recommendations [1,2,3], physician adherence to standardized protocols can vary in real-world settings. Such variability in implementation is a recognized global issue in DKA management and may contribute to minor differences in clinical outcomes between centers. Evaluating protocol adherence, however, was beyond the scope of this descriptive study and is an area for future research.

Potassium-related abnormalities frequently complicate clinical pictures both at presentation and during treatment. Moreover, potassium levels at admission play a role in the treatment plan and are found to be a predictor of LoS in hospitals in other regional studies [18,19]. A high incidence of both hyperkalemia and hypokalemia was observed in our study after treatment initiation. Hyperkalemia was identified in 23.6% of adult patients and 24.5% of pediatric patients, while hypokalemia was documented in 20.2% of adults and 24.5% of pediatric patients, both of which are comparable to other regional findings. A Saudi study by Batwa et al. observed hypokalemia in 24.5% of pediatric patients, while Al Thiabat et al. reported a similar rate of 24% in Jordan [13,19]. The rates of both hyperkalemia and hypokalemia reported in our study are in the same range as those reported in similar patient populations. This reinforces that electrolyte disturbances are a very common complication of DKA management and highlights the need for electrolyte monitoring to safely manage the predictable yet potentially dangerous potassium-related complications.

Organ dysfunction, particularly AKI, can both precipitate and result from DKA. Chronic cardiac or hepatic impairment was infrequently documented but may have contributed to acidosis severity in some adult cases. AKI occurred more frequently in adult patients (5.2%) than in pediatric patients (1.9%). Although these rates are lower than those reported in other regional studies [10,19], AKI occurrence remains clinically significant. The lower observed incidence in pediatric patients may reflect differences in ICU monitoring intensity and earlier fluid resuscitation. Pediatric patients at presentation exhibited slightly greater metabolic acidosis (lower bicarbonate levels and higher anion gaps) and a lower median creatinine clearance level (62.3 mL/min vs. 70.0 mL/min) compared to adult patients. This may suggest that renal function was proportionally more affected relative to baseline. A decrease in CrCl of this magnitude indicates relatively greater physiological renal compromise than the absolute values suggest, as children’s smaller muscle mass naturally leads to lower baseline serum creatinine levels. This observation aligns with the findings by Al Thiabat et al., who reported that severe acidosis and dehydration are identified as important risk factors for AKI in pediatric patients [19]. Moreover, Musoma et al. found that elevated serum creatinine levels and decreased urine output were significant predictors of mortality in pediatric patients with DKA [20]. Although the incidence of AKI was lower in our pediatric cohort, the condition remains clinically relevant in DKA due to its potential impact on outcomes. Hursh et al. found that 64% of children with DKA developed AKI, with nearly all cases occurring within the first 24 h of admission [21]. Their study identified severe acidosis, volume depletion, and elevated corrected sodium as key predictors, emphasizing that AKI may develop early and require vigilant monitoring even when incidence is low.

The findings of this study are of importance given the continuing increase in DM prevalence in Saudi Arabia. The increase in prevalence, especially among the youth, leads to an increase in DKA burden unless effective preventive measures are taken. As demonstrated in this study, the high admission rates were attributed to preventable causes such as non-compliance with therapy and infections. While the study did not directly assess patient literacy or educational interventions, the predominance of non-compliance and infections as precipitating factors highlights potential public health and clinical targets for reducing DKA recurrence. Indeed, comprehensive outpatient care can help strengthen the continuity of care and help decrease the burden on emergency and inpatient services. A survey-based study conducted in Makkah, Saudi Arabia, found that the overall awareness of patients and their caregivers regarding DKA is poor (67%), with only 16% linking infections as a risk factor and only 29.3% identifying missing insulin doses as a main cause of DKA [22]. Another study in the northern borders of Saudi Arabia found that 67% of participants had poor knowledge about DM, with only 26% identifying insulin deficiency as the main cause [23]. These results emphasize that efforts are needed to develop culturally sensitive educational programs for patients and their families. These programs should cover sick-day management, the importance of insulin therapy and continuity of diabetes care, and understanding early signs of DKA, especially among pediatric cases.

This study has some limitations that need to be noted. This is a descriptive study that relied on the accuracy of healthcare providers’ documentation in the electronic health records. Missing information was noted during the data collection stage; this led to instances of missing data, particularly for variables such as family history of diabetes, diabetes onset and duration, lactate level, chronic organ dysfunction, and pre-admission medication history. Because the study was designed as a descriptive analysis, no inferential statistical testing was performed. In addition, the relatively small sample size in both the adult and pediatric cohorts and the limited number of outcome events precluded the use of multivariable regression models at this stage to adjust for potential confounders such as DKA severity at presentation, comorbidities, and variations in treatment protocols. Consequently, the independent effect of age groups on outcomes like time to DKA resolution, length of stay, and ICU admission was not assessed. In this study, we did not investigate the history of previous DKA events, which have been reported by other studies as a risk factor for future DKA events. Our study retrieved data from two large hospitals in the Riyadh region, which may not be representative of other regions. Moreover, we have focused on acute hospital admissions, and we did not investigate different treatment strategies and adherence to clinical guidelines or long-term follow-up data. Post-discharge mortality data were not available from our retrospective records, and outcomes were therefore limited to in-hospital mortality events. Overall, given the retrospective and exploratory design of this study, the findings should be interpreted as descriptive associations rather than causal relationships.

## 5. Conclusions

This study described the clinical characteristics and outcomes of adult and pediatric patients with DKA in two tertiary hospitals in Saudi Arabia. Non-compliance with diabetes therapy and infections were the most frequently identified precipitating factors in patients with DM before the index DKA event. Potassium-related disturbances and AKI were notable complications that require careful monitoring in all patients. Pediatric cases appeared to have faster biochemical resolution but longer hospital stays and higher ICU admission rates. These findings are descriptive and should be interpreted with caution, as observed differences may reflect patient variability or random fluctuations. Further studies with larger samples and analytical designs are needed to confirm these observations and explore long-term outcomes.

## Figures and Tables

**Table 1:** Demographics and clinical characteristics of DKA patients (373 patients).

Variable	All	Adults	Pediatrics
Number of patients	373 (100.0)	267 (71.6)	106 (28.4)
**Age, years**	18.7 (14.4–31.0)	24.3 (17.5–39.2)	12.9 (11.3–13.9)
**Gender**			
Male	170 (45.6)	134 (50.2)	36 (34.0)
Female	203 (54.4)	133 (49.8)	70 (66.0)
**Body Mass Index (BMI), kg/m^2^**	21.6 (17.8–25.6)	22.7 (19.0–26.8)	18.2 (14.6–22.8)
**Family history of DM**			
Type 1	37 (9.9)	15 (5.6)	22 (20.8)
Type 2	46 (12.3)	26 (9.7)	20 (18.9)
Gestational	2 (0.5)	1 (0.4)	1 (0.9)
No history of DM	43 (11.5)	24 (9.0)	19 (17.9)
Not documented	258 (69.2)	205 (76.8)	53 (50.0)
**The onset of diabetes**			
Pre-existing diabetes	321 (86.1)	237 (88.8)	84 (79.2)
New onset of diabetes	52 (13.9)	30 (11.2)	22 (20.8)
**Type of DM**			
Type 1	304 (81.5)	198 (74.2)	106 (100.0)
Type 2	64 (17.2)	64 (24.0)	0 (0.0)
Other	5 (1.3)	5 (1.9)	0 (0.0)
**Comorbidities**			
Hypertension	48 (12.9)	48 (18.0)	0 (0.0)
Dyslipidemia	47 (12.6)	47 (17.6)	0 (0.0)
Asthma	11 (2.9)	7 (2.6)	4 (3.8)
Cardiovascular diseases	11 (2.9)	11 (4.1)	0 (0.0)
Stroke or TIA	9 (2.4)	9 (3.4)	0 (0.0)
Others	69 (18.5)	61 (22.8)	8 (7.5)
**Diabetes-related complications**			
Diabetic retinopathy	15 (4.0)	15 (5.6)	0 (0.0)
Diabetic nephropathy	10 (2.7)	10 (3.7)	0 (0.0)
Diabetic foot injury or amputation	8 (2.1)	8 (3.0)	0 (0.0)
Cardiovascular disease	6 (1.6)	6 (2.2)	0 (0.0)
Diabetic neuropathy	4 (1.1)	4 (1.5)	0 (0.0)
Cerebrovascular diseases	3 (0.8)	3 (1.1)	0 (0.0)
Peripheral arterial disease	1 (0.3)	1 (0.4)	0 (0.0)

Data are presented as the median (IQR) or the number of patients (%). Abbreviations: DKA: Diabetic Ketoacidosis; SD: Standard Deviation; IQR: Interquartile Range; DM: Diabetes Mellitus; TIA: Transient Ischemic Attack.

**Table 2:** Severity classification of DKA among patients.

Age and Severity	Adults			Pediatrics		
Variables	Mild	Moderate	Severe	Mild	Moderate	Severe
Number of patients	41 (15.4)	77 (28.8)	149 (55.8)	25 (23.6)	44 (41.5)	37 (34.9)
**Glucose, mmol/L**	18.0 (15.5–24.5)	23.40 (19.0–30.9)	25.10 (19.8–30.8)	21.60 (18.2–22.8)	23.10 (18.3–27.2)	26.10 (20.7–29.5)
**pH**	7.3 (7.27–7.33)	7.2 (7.20–7.29)	7.1 (7.00–7.17)	7.3 (7.24–7.28)	7.2 (7.10–7.20)	7.0 (6.9–7.1)
**Bicarbonate, mmol/L**	17.0 (16.0–17.0)	12.0 (10.0–13.0)	5.0 (5.0–7.0)	12.0 (10.0–14.0)	8.0 (6.5–9.0)	5.0 (4.0–6.0)
**Anion gap**	17.0 (15.0–20.0)	21.60 (19.0–24.0)	27.0 (23.0–32.0)	21.0 (18.0–23.0)	26.5 (21.0–29.0)	29.0 (26.0–31.0)
**Urine ketone, positive**	34 (81.9)	73 (94.8)	142 (95.3)	25 (100.0)	44 (100.0)	37 (100.0)

Data are presented as the median (IQR) or the number of patients (%). Abbreviations: DKA: Diabetic Ketoacidosis; IQR: Interquartile range.

**Table 3:** Reasons for admission and predisposing factors for DKA *.

Variable	All	Adults	Pediatrics
Number of patients	321 (100.0)	237 (73.8)	84 (26.2)
**Reason for admission**			
Non-compliance with therapy	187 (58.3)	146 (61.6)	41 (48.8)
Inadequate or insufficient insulin therapy	19 (5.9)	12 (5.0)	7 (8.3)
Insulin pump malfunction	9 (2.8)	8 (3.4)	1 (1.2)
Lack of insulin (out of supply)	3 (0.9)	3 (1.3)	0 (0.0)
Other or non-documented	103 (32.1)	68 (28.7)	35 (41.7)
**Predisposing factors**			
Infection of any kind	66 (20.6)	58 (24.5)	8 (9.5)
Acute pancreatitis	4 (1.2)	3 (1.3)	1 (1.2)
Severe burn/trauma	4 (1.2)	4 (1.7)	0 (0.0)
Alcohol abuse	4 (1.2)	4 (1.7)	0 (0.0)
Cerebrovascular accident (stroke or TIA)	3 (0.9)	4 (1.3)	0 (0.0)
Drug abuse	3 (0.9)	3 (1.3)	0 (0.0)
Myocardial infarction (MI)	2 (0.6)	2 (0.8)	0 (0.0)

Data are presented as the number of patients (%). Abbreviations: DKA: Diabetic Ketoacidosis; TIA: Transient Ischemic Attack. * Table represents patients with pre-existing diabetes only; new-onset diabetes cases were excluded from this analysis.

**Table 4:** Diabetes management prior to hospitalization.

Variable	All	Adults	Pediatrics
Number of patients	373 (100.0)	271 (71.6)	106 (28.4)
**Insulin injections or pens**	288 (77.2)	208 (77.9)	80 (75.5)
Rapid-acting insulin	271 (72.7)	200 (74.9)	71 (67.0)
Long-acting insulin	268 (71.8)	192 (71.9)	76 (71.7)
Ultra-long-acting insulin	3 (0.8)	3 (1.1)	0 (0.0)
Regular or short-acting insulin	8 (2.1)	3 (1.1)	5 (4.7)
Intermediate-acting insulin: NPH	2 (0.5)	1 (0.4)	1 (0.9)
**Insulin pump**	16 (4.3)	12 (4.5)	4 (3.8)
**Non-insulin hypoglycemic agents**	30 (8.0)	27 (10.1)	3 (2.8)
Biguanides	23 (6.2)	23 (8.6)	0 (0.0)
DPP-4i	7 (1.9)	7 (2.6)	0 (0.0)
Sulfonylureas	5 (1.3)	5 (1.9)	0 (0.0)
GLP-1RA	4 (1.1)	1 (0.4)	3 (2.8)
SGLT-2i	2 (0.5)	2 (0.7)	0 (0.0)
Thiazolidinediones	1 (0.3)	1 (0.4)	0 (0.0)
**Not applicable or not documented**	57 (15.3)	35 (13.1)	22 (20.8)

Data are presented as the number of patients (%). Abbreviations: GLP-1RA: glucagon-like peptide-1 receptor agonist; DPP-4i: dipeptidyl peptidase-4 inhibitors; SGLT-2i: sodium–glucose co-transporter-2 inhibitor.

**Table 5:** Baseline mental status and laboratory values at admission.

Variable	All	Adults	Pediatrics
Number of patients	373 (100.0)	267 (71.6)	106 (28.4)
**Mental status at admission**			
Alert	317 (85.0)	228 (85.4)	89 (84.0)
Alert/drowsy	53 (14.2)	36 (13.5)	17 (16.0)
Stupor/coma	3 (0.8)	3 (1.1)	0 (0.0)
**Laboratory values at admission**			
Glucose (finger stick), mmol/L	23.0 (17.1–28.4)	23.1 (16.7–29.5)	23.0 (18.3–27.0)
Glucose (lab), mmol/L	25.4 (19.1–31.1)	25.9 (19.6–32.4)	24.5 (16.9–28.8)
SrCr, umol/L	92.0 (74.0–119.0)	97.0 (76.0, 127.0)	81.0 (69.0–97.0)
CrCl, ml/min	67.2 (50.9–87.8)	70.0 (51.5, 89.5)	62.3 (46.8–83.2)
BUN, mmol/L	5.4 (4.0–6.8)	5.4 (4.1–7.0)	5.4 (3.8–6.3)
Potassium, mmol/L	5.0 (4.4–5.4)	5.0 (4.4–5.4)	4.9 (4.4–5.4)
Sodium, mmol/L	133.0 (130.0–136.0)	133.0 (130.0–136.0)	134.0 (131.0–137.0)
Corrected sodium, mmol/L	141.2 (138.2–144.0)	141.2 (138.0–144.0)	140.9 (138.4–144.1)
Chloride, mmol/L	99.0 (96.0–103.0)	99.0 (95.0–102.0)	100.0 (97.0–103.0)
Bicarbonate, mmol/L	10.0 (6.0–14.0)	10.0 (6.0–15.0)	8.0 (6.0–11.0)
Serum osmolality, mOsm/kg H_2_O	303.0 (292.0–312.0)	304.0 (293.0–316.0)	298.0 (291.0–307.0)
Anion gap	24.0 (19.0–29.0)	23.0 (19.0–28.0)	26.0 (21.0–30.0)
PH	7.2 (7.1–7.3)	7.2 (7.1–7.3)	7.2 (7.1–7.3)
Urine ketone, positive	355 (95.2)	249 (93.3)	106 (100.0)

Data are presented as the median (IQR) or the number of patients (%). Abbreviations: DKA: Diabetic Ketoacidosis; IQR: Interquartile range; SrCr: Serum Creatinine; CrCl: Creatinine Clearance.

**Table 6:** Clinical outcomes and complications for DKA patients.

Variable	All	Adults	Pediatrics
Number of patients	373 (100.0)	267 (71.6)	106 (28.4)
**Resolution of DKA during hospitalization**	307 (82.3)	224 (83.9)	83 (78.3)
Time to resolve DKA, hours	22.0 (14.7–34.6)	23.7 (16.2–36.9)	16.5 (11.7–25.8)
**Patient with one of following complications**			
Hyperkalemia (K > 5.2 mmol/L)	89 (23.9)	63 (23.6)	26 (24.5)
Hypokalemia (K < 3.3 mmol/L)	80 (21.4)	54 (20.2)	26 (24.5)
Hypoglycemia (glucose < 4.0 mmol/L)	32 (8.6)	26 (9.7)	6 (5.7)
Acute kidney injury	16 (4.3)	14 (5.2)	2 (1.9)
Pulmonary edema	2 (0.5)	2 (0.7)	0 (0.0)
Cerebral edema	2 (0.5)	0 (0.0)	2 (1.9)
Death	3 (0.8)	3 (1.1)	0 (0.0)
None	203 (54.4)	149 (55.8)	54 (50.9)
**Patients’ final admission status**			
Admitted to general wards only	230 (61.7)	185 (69.3)	45 (42.5)
Admitted to general wards and the ICU	76 (20.4)	34 (12.7)	42 (39.6)
Admitted to the ICU only	33 (8.8)	21 (7.9)	12 (11.3)
Discharged from the ED	34 (9.1)	27 (10.1)	7 (6.6)
**Length of stay**			
ED, hours	6.9 (3.9–14.6)	8.3 (4.9–16.4)	3.8 (2.6–6.9)
General wards, days (n = 306)	1.5 (1.0–2.8)	1.5 (1.0–3.0)	1.5 (1.0–2.3)
ICU, days (n = 109)	1.0 (0.6–1.6)	1.2 (0.5–2.2)	0.9 (0.6–1.4)
Full length of stay in the hospital, days (n = 373)	2.0 (1.3–3.3)	2.0 (1.3–3.5)	2.1 (1.5–3.0)

Data are presented as the median (IQR) or the number of patients (%). Abbreviations: DKA: Diabetic Ketoacidosis; IQR: Interquartile Range; ED: Emergency Department; ICU: Intensive Care Unit.

## Data Availability

All data supporting the findings of this study are presented within the article and its Supplementary Materials. Additional details or clarifications can be obtained from the corresponding author upon reasonable request.

## Supplementary Materials

The following supporting information can be downloaded at https://www.mdpi.com/article/10.3390/jcm14238505/s1. Table S1. Demographics and clinical characteristics of DKA patients based on the DM type (368 patients); Table S2. Severity classification of DKA among patients; Table S3. Reasons for admission and predisposing factors of DKA based on the DM type (368 patients); Table S4. Diabetes management prior to hospitalization based on the DM type (368 patients); Table S5. Baseline mental status and laboratory values at admission based on the DM type (368 patients); Table S6. Clinical outcomes and complications in DKA patients based on their DM type (368 patients).
