# Supplementary material for: Precipitating Factors, Complications, and Outcomes of Diabetic Ketoacidosis (DKA) in Adults and Pediatrics: A Descriptive Study from Two Tertiary Centers in Riyadh, Saudi Arabia
Source: J Clin Med. 2025 Nov 30;14(23):8505. doi: 10.3390/jcm14238505 (PMC12693036; doi:10.3390/jcm14238505)
Supplement: Supplementary file 1 [file jcm-14-08505-s001.zip › jcm-3978264-supplementary.pdf]

Precipitating Factors, Complications, and Outcomes of Diabetic Ketoacidosis (DKA) in Adults and Pediatrics: A Descriptive Study from Two Tertiary Centers in Riyadh, Saudi Arabia

Table S1. Demographics and clinical characteristics of DKA patients based on the type of DM (368 patients).

Variable	All	T1DM	T2DM
Number of patients	368 (100.0)	304 (82.6)	64 (17.4)
**Age, years**	18.6 (14.4 – 31.0)	16.7 (13.8 – 23.0)	55.2 (41.5 – 46.2)
**Gender**			
Male	169 (45.9)	133 (43.8)	36 (56.3)
Female	199 (54.1)	171 (56.3)	28 (43.8)
**Body Mass Index (BMI), kg/m²**	21.6 (17.8 – 25.6)	20.9 (17.0 – 24.9)	25.5 (22.8 – 31.3)
**Family history of DM**			
Type 1	36 (9.8)	35 (11.5)	1 (1.6)
Type 2	46 (12.5)	40 (13.2)	6 (9.4)
Gestational	2 (0.5)	2 (0.7)	0 (0.0)
No history of DM	43 (11.7)	40 (13.2)	3 (4.7)
Not documented	254 (69.0)	200 (65.8)	54 (84.4)
**The onset of diabetes**			
Pre-existing diabetes	319 (86.7)	263 (86.5)	56 (87.5)
New onset of diabetes	49 (13.3)	41 (13.5)	8 (12.5)
**Comorbidities**			
Hypertension	48 (13.0)	12 (3.9)	36 (56.3)
Dyslipidemia	47 (12.8)	17 (5.6)	30 (46.9)
Asthma	11 (3.0)	7 (2.3)	4 (6.3)
Cardiovascular diseases	11 (3.0)	3 (1.0)	8 (12.5)
Stroke or TIA	9 (2.4)	2 (0.7)	7 (10.9)
Others	67 (18.2)	48 (15.8)	19 (29.7)
**Diabetes-related complications**			
Diabetic retinopathy	15 (4.1)	7 (2.3)	8 (12.5)
Diabetic nephropathy	10 (2.7)	7 (2.3)	3 (4.7)
Diabetic foot injury or amputation	8 (2.2)	4 (1.3)	4 (6.3)
Cardiovascular disease	6 (1.6)	2 (0.7)	4 (6.3)
Diabetic neuropathy	4 (1.1)	2 (0.7)	2 (3.1)
Cerebrovascular diseases	3 (0.8)	1 (0.3)	2 (3.1)
Peripheral arterial disease	1 (0.3)	0 (0.0)	1 (1.6)

Data are presented as the median (IQR), or the number of patients (%).

Five patients had other types of diabetes, and were excluded from the analysis in this table.

Abbreviations: DKA: Diabetic Ketoacidosis; SD: Standard Deviation; IQR: Interquartile Range; DM: Diabetes Mellitus; TIA: Transient Ischemic Attack.

Precipitating Factors, Complications, and Outcomes of Diabetic Ketoacidosis (DKA) in Adults and Pediatrics: A Descriptive Study from Two Tertiary Centers in Riyadh, Saudi Arabia

Table S2. Severity classification of DKA among patients.

Age & Severity	Adults			Pediatrics		
**Variables**	**Mild**	**Moderate**	**Severe**	**Mild**	**Moderate**	**Severe**
Number of patients	41 (15.4)	77 (28.8)	149 (55.8)	25 (23.6)	44 (41.5)	37 (34.9)
**Glucose, mmol/L**	18.0 (15.5 – 24.5)	23.40 (19.0 – 30.9)	25.10 (19.8 – 30.8)	21.60 (18.2 – 22.8)	23.10 (18.3 – 27.2)	26.10 (20.7 – 29.5)
**pH**	7.3 (7.27 – 7.33)	7.2 (7.20 – 7.29)	7.1 (7.00 – 7.17)	7.3 (7.24 – 7.28)	7.2 (7.10 – 7.20)	7.0 (6.90 – 7.10)
**Bicarbonate, mmol/L**	17.0 (16.0 – 17.0)	12.0 (10.0 – 13.0)	5.0 (5.0 – 7.0)	12.0 (10.0 – 14.0)	8.0 (6.5 – 9.0)	5.0 (4.0 – 6.0)
**Anion gap**	17.0 (15.0 – 20.0)	21.60 (19.0 – 24.0)	27.0 (23.0 – 32.0)	21.0 (18.0 – 23.0)	26.5 (21.0–29.0)	29.0 (26.0–31.0)
**Urine Ketone, positive**	34 (81.9)	73 (94.8)	142 (95.3)	25 (100.0)	44 (100.0)	37 (100.0)
**Type of DM***						
T1DM (n=304)	28 (14.1)	53 (26.8)	117 (59.1)	25 (23.6)	44 (41.5)	37 (34.9)
T2DM (n=64)	12 (18.8)	22 (34.4)	30 (46.8)	0 (0.0)	0 (0.0)	0 (0.0)

Data are presented as the median (IQR), or the number of patients (%).
* Five patients had other types of diabetes, and were excluded from this analysis.
Abbreviations: DKA: Diabetic Ketoacidosis; IQR: Interquartile range; DM: Diabetes Mellitus.

Table S3. Reasons for admission and predisposing factors for DKA based on the type of DM (319 patients).

Variable	All	T1DM	T2DM
Number of patients	319 (100.0)	263 (82.4)	56 (17.6)
**Reason for admission**			
Non-compliance with therapy	186 (58.3)	157 (59.7)	29 (51.8)
Inadequate or insufficient insulin therapy	19 (6.0)	16 (6.1)	3 (5.4)
Insulin pump malfunction	9 (2.8)	8 (3.0)	1 (1.8)
Lack of insulin (out of supply)	3 (0.9)	3 (1.1)	0 (0.0)
Other or non-documented	102 (31.9)	78 (29.6)	24 (42.9)
**Predisposing factors**			
Infection of any kind	65 (20.4)	49 (18.6)	16 (28.6)
Acute pancreatitis	4 (1.3)	4 (1.5)	0 (0.0)
Severe burn/trauma	4 (1.3)	4 (1.5)	0 (0.0)
Alcohol abuse	4 (1.3)	2 (0.8)	2 (3.6)
Cerebrovascular accident (stroke or TIA)	3 (0.9)	0 (0.0)	3 (5.4)
Drug abuse	3 (0.9)	3 (1.1)	0 (0.0)
Myocardial infarction (MI)	2 (0.6)	0 (0.0)	2 (3.6)

Data are presented as the number of patients (%).
Table represents patients with pre-existing diabetes only; new-onset diabetes cases were excluded from this analysis.
Five patients had other types of diabetes, and were excluded from the analysis in this table.
Abbreviations: DKA: Diabetic Ketoacidosis; TIA: Transient Ischemic Attack.

Precipitating Factors, Complications, and Outcomes of Diabetic Ketoacidosis (DKA) in Adults and Pediatrics: A Descriptive Study from Two Tertiary Centers in Riyadh, Saudi Arabia

Table S4. Diabetes management prior to hospitalization based on the type of DM (368 patients).

Variable	All	T1DM	T2DM
Number of patients	368 (100.0)	304 (82.6)	64 (17.4)
**Insulin injections or pens**	286 (77.7)	244 (80.3)	42 (65.6)
Rapid-acting insulin	269 (73.1)	231 (76.0)	38 (59.4)
Long-acting insulin	266 (72.3)	229 (75.3)	37 (57.8)
Ultra-long-acting insulin	3 (0.8)	3 (1.0)	0 (0.0)
Regular or short-acting insulin	8 (2.2)	6 (2.0)	2 (3.1)
Intermediate-acting insulin: NPH	2 (0.5)	2 (0.7)	0 (0.0)
**Insulin pump**	16 (4.3)	15 (4.9)	1 (1.6)
**Non-insulin hypoglycemic agents**	30 (8.2)	8 (2.6)	22 (34.4)
Biguanides	23 (6.3)	5 (1.6)	18 (28.1)
DPP-4i	7 (1.9)	1 (0.3)	6 (9.4)
Sulfonylureas	5 (1.4)	0 (0.0)	5 (7.8)
GLP-1RA	4 (1.1)	3 (1.0)	1 (1.6)
SGLT-2i	2 (0.5)	1 (0.3)	1 (1.6)
Thiazolidinediones	1 (0.3)	0 (0.0)	1 (1.6)
**Not applicable or not documented**	54 (14.7)	44 (14.5)	10 (15.6)

Data are presented as the number of patients (%).
Five patients had other types of diabetes, and were excluded from the analysis in this table.
Abbreviations: GLP-1RA: glucagon-like peptide-1 receptor agonist; DPP-4i: dipeptidyl pepti-dase-4 inhibitors; SGLT-2i: sodium–glucose co-transporter-2 inhibitor.

Table S5. Baseline mental status and laboratory values at admission based on the type of DM (368 patients).

Variable	All	T1DM	T2DM
Number of patients	368 (100.0)	304 (82.6)	64 (17.4)
**Mental status at admission**			
Alert	313 (85.1)	263 (86.5)	50 (78.1)
Alert/drowsy	52 (14.1)	39 (12.8)	13 (20.3)
Stupor/coma	3 (0.8)	2 (0.7)	1 (1.6)
**Laboratory values at admission**			
Glucose (finger stick), mmol/L	23.0 (17.3 – 28.4)	23.0 (16.4 – 28.2)	24.3 (19.6 – 31.0)
Glucose (lab), mmol/L	25.4 (19.1 – 31.1)	25.3 (18.3 – 30.7)	26.9 (20.8 – 33.8)
SrCr, umol/L	92.0 (74.0 – 119.0)	88.5 (73.0 – 112.0)	120.5 (92.5 – 153.5)
CrCl, ml/min	66.7 (50.5 – 87.6)	71.3 (54.4 – 90.1)	48.2 (35.0 – 70.0)
BUN, mmol/L	5.4 (4.0 – 6.8)	5.3 (3.9 – 6.3)	7.2 (5.2 – 11.1)
Potassium, mmol/L	5.0 (4.4 – 5.4)	5.0 (4.5 – 5.4)	5.1 (4.4 – 5.4)
Sodium, mmol/L	133.0 (130.0 – 136.0)	133.5 (130.0 – 136.0)	132.0 (128.0 – 135.0)
Corrected sodium, mmol/L	141.2 (138.2 – 144.1)	141.3 (138.4 – 144.2)	140.8 (137.8 – 143.3)
Chloride, mmol/L	99.0 (96.0 – 103.0)	100.0 (96.0 – 103.0)	98.0 (93.0 – 102.0)
Bicarbonate, mmol/L	10.0 (6.0 – 13.0)	9.4 (6.0 – 13.0)	10.0 (5.5 – 15.0)
Serum osmolality, mOsm/kg H_2O	303.0 (293.0 – 312.0)	302.0 (292.0 – 309.0)	310.0 (300.0 – 321.0)
Anion gap	24.0 (19.0 – 29.0)	24.0 (19.0 – 29.0)	23.0 (18.5 – 28.0)
PH	7.2 (7.1 – 7.3)	7.2 (7.1 – 7.3)	7.2 (7.1 – 7.3)
Urine ketone, positive	351 (95.4)	291 (95.7)	60 (93.8)

Data are presented as the median (IQR) or the number of patients (%).
Five patients had other types of diabetes, and were excluded from the analysis in this table.
Abbreviations: DKA: Diabetic Ketoacidosis; IQR: Interquartile range; SrCr: Serum Creatinine; CrCl: Creatinine Clearance.

Precipitating Factors, Complications, and Outcomes of Diabetic Ketoacidosis (DKA) in Adults and Pediatrics: A Descriptive Study from Two Tertiary Centers in Riyadh, Saudi Arabia

Table S6. Clinical outcomes and complications for DKA patients based on the type of DM (368 patients).

Variable	All	T1DM	T2DM
Number of patients	319 (100.0)	304 (82.6)	64 (17.4)
**Resolution of DKA during hospitalization**	304 (82.6)	249 (81.9)	55 (85.9)
Time to resolve DKA, hours	21.9 (14.8 – 34.3)	21.4 (14.3 – 33.7)	24.0 (16.0 – 38.0)
**Patient with one of following complications**			
Hyperkalemia (K > 5.2 mmol/L)	88 (23.9)	78 (25.7)	10 (15.6)
Hypokalemia (K < 3.3 mmol/L)	79 (21.5)	65 (21.4)	14 (21.9)
Hypoglycemia (glucose < 4.0 mmol/L)	32 (8.7)	25 (8.2)	7 (10.9)
Acute kidney injury	16 (4.3)	9 (3.0)	7 (10.9)
Pulmonary edema	2 (0.5)	0 (0.0)	2 (3.1)
Cerebral edema	2 (0.5)	2 (0.7)	0 (0.0)
Death	3 (0.8)	0 (0.0)	3 (4.7)
None	200 (54.3)	163 (53.3)	37 (57.8)
**Patients' final admission status**			
Admitted to general wards only	226 (61.4)	176 (57.9)	50 (78.1)
Admitted to general wards and the ICU	75 (20.4)	68 (22.4)	7 (10.9)
Admitted to the ICU only	33 (9.0)	27 (8.9)	6 (9.4)
Discharged from the ED	34 (9.2)	33 (10.9)	1 (1.6)
**Length of stay**			
ED, hours	6.9 (3.9 – 14.7)	5.9 (3.7 – 14.3)	9.9 (6.6 – 15.7)
General wards, days (n=301)	1.5 (1.0 – 2.8)	1.4 (0.9 – 2.4)	2.7 (1.5 – 4.0)
ICU, days (n=108)	1.0 (0.6 – 1.6)	0.9 (0.6 – 1.5)	1.6 (0.7 – 2.5)
Full length of stay in the hospital, days (n=368)	2.0 (1.3 – 3.2)	1.8 (1.2 – 3.0)	2.9 (1.8 – 5.0)

Data are presented as the median (IQR) or the number of patients (%).

Five patients had other types of diabetes, and were excluded from the analysis in this table.

Abbreviations: DKA: Diabetic Ketoacidosis; IQR: Interquartile Range; ED: Emergency Depart-ment; ICU: Intensive Care Unit.
